# Integrated Genomic and Transcriptomic Analysis reveals key genes for predicting dual-phenotype Hepatocellular Carcinoma Prognosis

**DOI:** 10.7150/jca.56005

**Published:** 2021-03-19

**Authors:** Yaobang Wang, Xi Wang, Xiaoliang Huang, Jie Zhang, Junwen Hu, Yapeng Qi, Bangde Xiang, Qiuyan Wang

**Affiliations:** 1 Guangxi Key Laboratory for Genomic and Personalized Medicine, Guangxi Medical University, Nanning, Guangxi Zhuang Autonomous Region, China.; 2 Guangxi Collaborative Innovation Center for Genomic and Personalized Medicine, Guangxi Medical University, Nanning, Guangxi Zhuang Autonomous Region, China.; 3 Department of Clinical Laboratory. First Affiliated Hospital of Guangxi Medical University, Nanning, Guangxi Zhuang Autonomous Region, China.; 4 Department of Hepatobiliary Surgery, Guangxi Medical University Cancer Hospital, Guangxi Zhuang Autonomous Region, China.

**Keywords:** CXCL9, dual-phenotype hepatocellular carcinoma, whole exome sequencing, RNA sequencing, prognosis

## Abstract

Dual-phenotype hepatocellular carcinoma (DPHCC) expresses both hepatocyte and cholangiocyte markers, and is characterized by high recurrence and low survival rates. The underlying molecular mechanisms of DPHCC pathogenesis are unclear.

We performed whole exome sequencing and RNA sequencing of three subtypes of HCC (10 DPHCC, 10 CK19-positive HCC, and 14 CK19-negative HCC), followed by integrated bioinformatics analysis, including somatic mutation analysis, mutation signal analysis, differential gene expression analysis, and pathway enrichment analysis. Cox proportional hazard regression analyses were applied for exploring survival related characteristics.

We found that mutated genes in DPHCC patients were associated with carcinogenesis and immunity, and the up-regulated genes were mainly enriched in transcription-related and cancer-related pathways, and the down-regulated genes were mainly enriched in immune-related pathways. CXCL9 was selected as the hub gene, which is associated with immune cells and survival prognosis. Our results showed that low CXCL9 expression was significantly associated with poor prognosis, and its expression was significantly reduced in DPHCC samples.

In conclusion, we explored the molecular mechanisms governing DPHCC development and progression and identified CXCL9, which influences the immune microenvironment and prognosis of DPHCC and might be new clinically significant biomarkers for predicting prognosis.

## Introduction

Liver cancer is one of the most common malignant tumors in the world 1. It originates in the liver, and frequently occurs in chronic liver diseases and cirrhosis 2. Hepatocellular carcinoma (HCC) and intrahepatic cholangiocarcinoma (ICC) are the most common pathological types of primary liver cancer, accounting for approximately 70% and 15% of liver cancer patients, respectively 3. Combined hepatocellular cholangiocarcinoma (CHC) 4 is a rare subtype that accounts for 1.0-14.2% of primary liver cancer 5, 6. Different from CHC, dual-phenotype hepatocellular carcinoma (DPHCC) is a new subtype of HCC 7. Previously, we found that DPHCC had a higher rate of postoperative recurrence and a lower survival rate than non-DPHCC 8.

CHC is characterized by the presence of both classical HCC and ICC components within a single nodule, often showing transitional or intermediate regions between the two 7. DPHCC shows typical morphological features of HCC and more than 15% of its tumor cells strongly express both hepatocyte and cholangiocyte markers. The pathological diagnosis of DPHCC is: (1) Immunohistochemically, more than 15% of the tumor cells, at least one hepatocyte marker (such as Hep Par 1) shows strong positive expression, and mainly diffuse distribution; (2) More than 15% of tumor cells, at least one cholangiocyte marker (such as CK19), and at least one hepatocyte marker (such as Hep par 1) are co-expressed. If the tumor tissue contains any component of HCC and intrahepatic cholangiocarcinoma, no matter whether there is a transition zone between these components or the tissue shows that tumor cells do not express markers of both hepatocytes and cholangiocarcinoma, these patients cannot be diagnosed as DPHCC 7, 8. While the incidence rate of DPHCC is much lower than that of CHC 9, DPHCC is more aggressive and exhibits a worse postoperative prognosis.

One of the typical characteristics of DPHCC is the positive expression of CK19, which is a marker of cancer stem cells (CSCs), and plays an important role in the formation, development, and maintenance of tumors 10-12. As CK19 promotes angiogenesis and tumor cell invasiveness, CK19 positive HCC (CK19+HCC) is more malignant and has a worse prognosis than CK19 negative HCC (CK19-HCC) 13-17. While DPHCC exhibits CK19+HCC, it is different from CK19+HCC, which is defined as the presence of moderate or strong expression of CK19 in membranous and/or cytoplasmic in 5% or more of tumor cells 18.

Compared to traditional HCC, DPHCC has a higher rate of vascular invasion, recurrence, and a worse prognosis, and exploring the molecular mechanisms underlying DPHCC will benefit the development of treatment strategies.

Sequencing technology enables the discovery of genetic alterations that contribute to the diagnosis and treatment of human cancers, as well as the provision of new targeted therapies 19. In addition to the effects of gene products, the persistent accumulation of somatic genomic alterations is also related to tumorigenesis 20-23. However, if most of the genetic alterations occur in passenger genes, they have little effect on the occurrence of cancer, and a small number of cancer driver genes involved in key signaling pathways of hepatocarcinogenesis have mutations, resulting in carcinogenesis 24. Whole exome sequencing (WES) has been used to identify the mutant cancer-driving genes in HCC 24-28, and TP53 and CTNNB1 are the two most commonly mutated genes in HCC 29, 30. Activation of the PI3K/Akt/mTOR pathway can enhance the growth, survival, and metabolism of cancer cells 31, and about 5-10% of HCC cases show activation 32.

Although genomic studies on traditional HCC have been thorough, genomic studies on DPHCC are rarely reported. The aim of the present study was to use WES and RNA sequencing to elucidate the genomic profile of DPHCC.

## Materials and Methods

The study protocol was approved by the Research Ethics Review Board of Guangxi Medical University Cancer Hospital (LW2020046). Before resection, all patients provided written informed consent for their data to be used for scientific research.

### Sample source

Thirty-four tumor samples were obtained from patients with HCC who underwent radical hepatectomies at Guangxi Medical University Cancer Hospital. Tissue samples from patients meeting the following criteria were used for analysis: (1) Pathologically diagnosed patients with HCC; (2) Patients with Child-Pugh grades A and B; (3) There was no residual tumor or portal vein tumor thrombus on imaging after radical resection, and alpha-fetoprotein level decreased to normal within 2 months. Patients who had any other malignancy or whose tumor tissue samples had not been paraffin embedded were excluded. Thirty-four patients met the criteria.

### Immunofluorescence double-staining

HCC tissues were fixed with 10% neutral formalin at room temperature for 24 h. After dehydration, transparency, and paraffin embedding, serial paraffin sections (4 µm thick) were generated, followed by deparaffinization and rehydration. Sections were incubated with anti-Hep Par-1 and CK19 antibodies simultaneously. Then, sections were stained with goat anti-rabbit secondary antibody labeled with tetramethylrhodamine-5(6)-isothiocyanate (TRITC; 111-026-045, Jackson ImmunoResearch, USA) and goat anti-mouse secondary antibody labeled with fluorescein isothiocyanate (FITC; 111-026-045, Jackson ImmunoResearch, USA) under dark conditions at 37 °C for 1 h. Cells were then counterstained with 4',6-diamidino-2-phenylindole (DAPI; C0065, Solarbio, China) for 2 min to localize the nuclei. Finally, sections were viewed under a fluorescence microscope.

### DNA extraction and library construction

HiPure Tissue DNA Mini kit (Magen, Guangzhou, China) was used to extract DNA from liver tissue. 10 mg of liver tissue was taken from the patient to obtain tissue DNA. Ultrasound fragmentation of DNA was performed to build a DNA library. After PCR amplification, sequencing was performed.

### Whole exome sequencing

Genomic DNA was extracted from fresh frozen tumors, and high-throughput, high-depth sequencing was performed on an Illumina sequencing platform (Illumina, USA), according to the manufacturer's instructions. Fastq files were obtained after sequencing, bases were identified, and quality control, such as removing junction sequences and low quality and short length reads, was performed. Using the MEM algorithm of BWA (v0.7.17, default parameter) 33, the standard sequence was aligned with the filtered data, and the reference sequence was hg19 (http://hgdownload.soe.ucsc.edu/goldenPath/hg19/bigZips/) to obtain the preliminary alignment results in Bam format. Picard software (https://broadinstitute.github.io/picard/) was used to calculate the proportion of redundant sequences caused by PCR amplification during exon capture experiments for each sample. GATK standard processing 34 was used to correct the base quality and errors caused by insertion and deletion in Bam format. SNV/ In Del were found using the GATK-MuTect2 detection method 35. The filtering method for all SNV/In Del loci was to set parameters for direct filtering and retain loci that meet the following conditions: public databases (gnomAD database, 1000 Genomes database, and ExAC database) with frequencies below 0.01. The vcf files were obtained after filtering, all SNV/In Del loci were compared and analyzed with the latest published population database by ANNOVAR 36, the mutation frequencies of these SNV/In Del loci in the population database were evaluated, and the SNV/In Del loci were classified and screened. We obtained average 30.678 GB of total clean data yield per sample, with an average total clean data read of 245060264.7, while the mean insert sizes were in the range of 115.35~ 164.967 base pairs. On average 99.08% of reads were mapped, 98.24% were properly paired, and we had less than 0.15% of average singletons. The average capture efficiency rate per sample on target region was 78.36%, the average capture efficiency rate on or near + - 150 target region was 79.36%, and the average capture efficiency rate on or near + - 500 target region was 80.41%. The average coverage of official target region per sample was at least 20X > 95.75%, the average coverage was at least 10X > 98.83%, and the average coverage was at least 2X > 99.86%. Fisher's exact test was used to determine whether the frequency of each locus in the DPHCC/CK19+HCC group was significantly different to that in the CK19-HCC group (according to P < 0.05) (CK19-HCC group was the control group).

### Mutation signature analysis

Mutation signature analysis deconvolutes cancer somatic mutation counts, separated by mutation contexts or biologically meaningful subgroups, into a set of characteristic signatures and infers the pattern of each of the discovered signatures across samples. We investigated the mutational spectrum of 96 subtypes of three-base context of mutations, considering six substitution patterns (C > A, C > G, C > T, T > A, T > C, and T > G) for all WES mutation data. MutationalPatterns 37 used the Non-negative Matrix Factorization (NMF) algorithm to extract signatures. The other two R packages: “deconstructSigs” 38 and “SomaticSignatures” 39 were used to further validate the extracted signatures.

The “deconstructSigs” uses an iterative approach to calculate the combination of Catalogue of Somatic Mutations in Cancer (COSMIC) 40 signatures that best approximate a tumor's mutational spectrum. Therefore, “deconstructSigs” can analyze individual samples and the results are more comparable to previous studies. We use the “mut.to.sigs.input” function was used to construct the appropriate input data structure. “whichSignatures” were then used to determine which of the COSMIC signatures were present in the tumor samples and their contribution to the total mutation spectrum 38. This function uses an iterative algorithm to find the combination and relative weights of signatures that best match each mutation spectrum.

Unlike “deconstructSigs”, “SomaticSignatures” takes a cohort of tumor's mutational spectra and uses either principal component analysis or NMF to identify signatures that are present within the cohort, and their contribution to each tumor's mutational spectrum. We used the “mutationContext” function in the “SomaticSignatures” package to extract the 3-nucleotide mutational context of each Single Nucleotide Variant (SNV). This function compares the loci of each SNV with the corresponding reference genome to identify nucleotides immediately 3′ and 5′ of the SNV. The “motifMatrix” function calculates the frequency of each of the 96 alteration types. To determine how many signatures we expected to identify, we ran the “assessNumberSignatures”. The “identifySignatures” method was used to decompose the mutational spectra of individuals in DPHCC into novel signatures, using the NMF option.

The mutation data of 154 Asian hepatocellular carcinomas were downloaded from The Cancer Genome Atlas (TCGA) and their mutation signatures were extracted with the “SomaticSignatures” R package for comparison with those of DPHCC patients.

### Copy number analysis

We identified copy number variants (CNVs) with default parameters of CNVkit 41 based on exome-sequencing data to analyze the copy number state of each tumor. We compared DPHCC tissues with CK19-HCC tissues with CNVkit. Based on previous studies, we used a log_2_ ratio cut-off of +/-0.25 to define copy number gain/loss 42. The cnvkit.py scatter/heatmap in CNVkit was used to plot DPHCC copy number calls across the genome with default setting.

### RNA extraction and cDNA library construction

HiPure Universal RNA Mini Kit (Magen, Guangzhou, China) was used to extract RNA from liver tissue. After obtaining the RNA from the liver tissues of patients and ensuring the quality of the extracted RNA was qualified, the cDNA library was constructed. We identified the polyA tail with Oligo dT (magnetic beads with a T sequence) to enrich the mRNA, then amplified it with random primer. Add an “A” base to its 3' end, attach adapter, and sequence.

### RNA sequencing

RNA was extracted from frozen tumors and sequenced on the Illumina platform. As raw reads often contain jointed and low-quality sequences, the original data need to be filtered. FastQC software was used to evaluate the quality of raw sequencing data for each sample, and Trimmomatic was used to remove raw reads containing joints, duplications, and lower sequencing quality, to obtain high-quality sequence data (clean reads). Hisat2 software 43 was used to align the sequencing data of each sample after quality control to the human reference genome. The transcripts were assembled and the expression levels were predicted using Stringtie software 44. DESeq2 45 was used for differential analysis. The P-value is the probability of rejecting or failing to reject the null hypothesis (H0) 46. H0 is the hypothesis that there is no difference between two groups for a specific variable. The P value measures the strength of evidence against H0 47. The smaller the P value, the stronger the evidence against H0. Fisher suggested 5% (α=0.05) level could be used for concluding that fairly strong evidence exists against H0. Based on the definition of statistically significant differences and the method of referring to a large number of relevant literatures 48-53, we also defined the differential genes as | Fold Change | > 1.5, P-value < 0.05.

### Gene ontology (GO) and KEGG functional enrichment analysis of DEGs

DAVID (https://david.ncifcrf.gov/) and KOBAS 3.0 were used for online analysis, which included data annotation, visualization, integration, extraction of important biological information 54, 55. GO enrichment analysis 56 is a major tool in bioinformatics for enrichment analysis of gene sets, which can identify the potential biological processes of target genes. GO mainly includes three levels, namely biological process, cellular components, and molecular functions. KEGG 57 is a comprehensive knowledge base for both functional interpretation of genomic information. Significant enrichment was defined as having a P-value < 0.05.

### Protein-protein interaction (PPI) network analysis

We constructed the PPI network information of DEGs of DPHCC using a search tool (STRING; http://stringdb.org, Version 11.0) 58 to retrieve interacting genes. Cytoscape software (3.7.2) 59 was used to construct PPI networks and to analyze functional interactions between proteins. After referring to the literature of similar studies 48, 60, 61, we also set all parameters by default. The Cytoscape software plug-in tool cytoHubba 62 was used to clarify the biological significance of gene modules (sub-networks) in HCC. P < 0.05 was considered statistically significant.

### Survival and correlation analysis between gene expression and immune infiltration level

OncoLnc 63 (http://www.oncolnc.org/) is an online tool used for interactively exploring the survival data of 8647 patients from 21 cancer studies in the Cancer Genome Atlas (TCGA), as well as mRNA and miRNA RNA-Seq expression data from TCGA. This tool allows the generation of Kaplan-Meier maps stratified by gene expression levels. Log-rank P values in survival analysis were recorded. The OncoLnc tool was used for validation of gene overall survival analysis. Patients were divided into two groups for comparison, based on the lowest and highest quartiles of gene expression, as recommended by web tools. The landmark analysis 64 was used to correct for the bias inherent in the analysis of time-to-event outcomes between groups determined during study follow-up. Tumor Immune Estimation Resource (TIMER) 65 (https://cistrome.shinyapps.io/timer/) is a comprehensive resource for systematical analysis of immune infiltrates across diverse cancer types, and the abundance of six immunes infiltrate (B cells, CD4+ T cells, CD8+ T cells, neutrophils, macrophages, and dendritic cells) were estimated with the TIMER algorithm. Based on previously applied deconvolution methods 66, TIMER reanalyzed gene expression data, including 10,897 samples from 32 cancer types from the Cancer Genome Atlas (TCGA), to estimate six tumor-infiltrating immune cells (TIICs) subgroups (B cells, CD4 T cells, CD8 T cells, macrophages, neutrophils, and dendritic cells). TIMER selected informative genes that were negatively correlated with tumor purity (percentage of malignant cells in tumor tissue) for each cancer type 67 and applied constrained least squares fitting to the selected gene expression to predict the abundance subset of six TIICs 65. The webservers and analysis tools of TIMER were used for the correlation analysis between gene expression and immune infiltration levels.

### Statistical analysis

SPSS 23.0 and GraphPad Prism 5 were used for statistical analysis, and a P-value < 0.05 was defined as statistically significant. Fisher's chi-square test (2-sided) was used to evaluate the significance of the differences between groups. The overall survival rate was calculated using R statistical software packages, such as “survival”, “survminer”, and “ggplot2”, and the difference between survival curve groups was tested by log-rank sum. Univariate and multivariate analysis of Cox proportional risk regression model was used to identify independent predictors of overall survival after hepatectomy in patients with HCC. The predictive value score, including sensitivity and specificity of risk, was assessed by receiver operating characteristic curve analysis.

## Results

### Immunofluorescence double-staining

**Figure 1:**
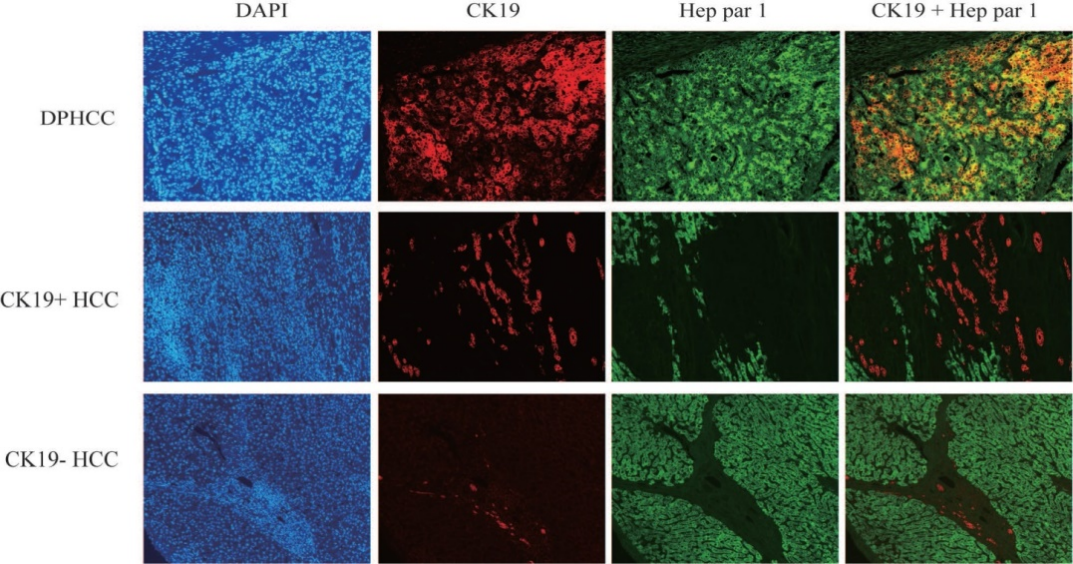
Immunofluorescence double-staining of hepatic tumor tissues from patients with DPHCC, CK19+HCC and CK19-HCC for the cholangiocytic marker CK19 (red) and hepatocyte marker Hep Par-1 (green). The cells with overlapping red and green colors are dual-phenotype cells.

Three subtypes of hepatocellular carcinoma, namely DPHCC, CK19+HCC, and CK19-HCC, showed reddish cholangiocyte marker CK19 and green hepatocyte marker Hep par1 by immunofluorescence double staining. DPHCC simultaneously expressed the cholangiocyte marker CK19 (red) and the hepatocyte marker Hep par1 (green), and both markers co-expressed more than 15% of tumor cells (green and red overlap more than 15%). CK19+HCC expressed the cholangiocyte marker CK19 in tumor cells (>5% in red), while CK19-HCC only expressed the hepatocyte marker Hep par1 (green) (Figure 1).

### Clinical characteristics

A total of 34 clinical characteristics of hepatocellular carcinoma patients, including 10 DPHCC, 10 CK19+HCC and 14 CK19-HCC patients are summarized. Corresponding patient demographic and clinical characteristics such as age, gender, liver cirrhosis, tumor size, tumors number, Edmondson grade, BCLC stage, microvascular invasion (MVI), distant metastasis, and AFP level is shown in Table 1.

### Somatic mutations in HCCs

**Figure 2:**
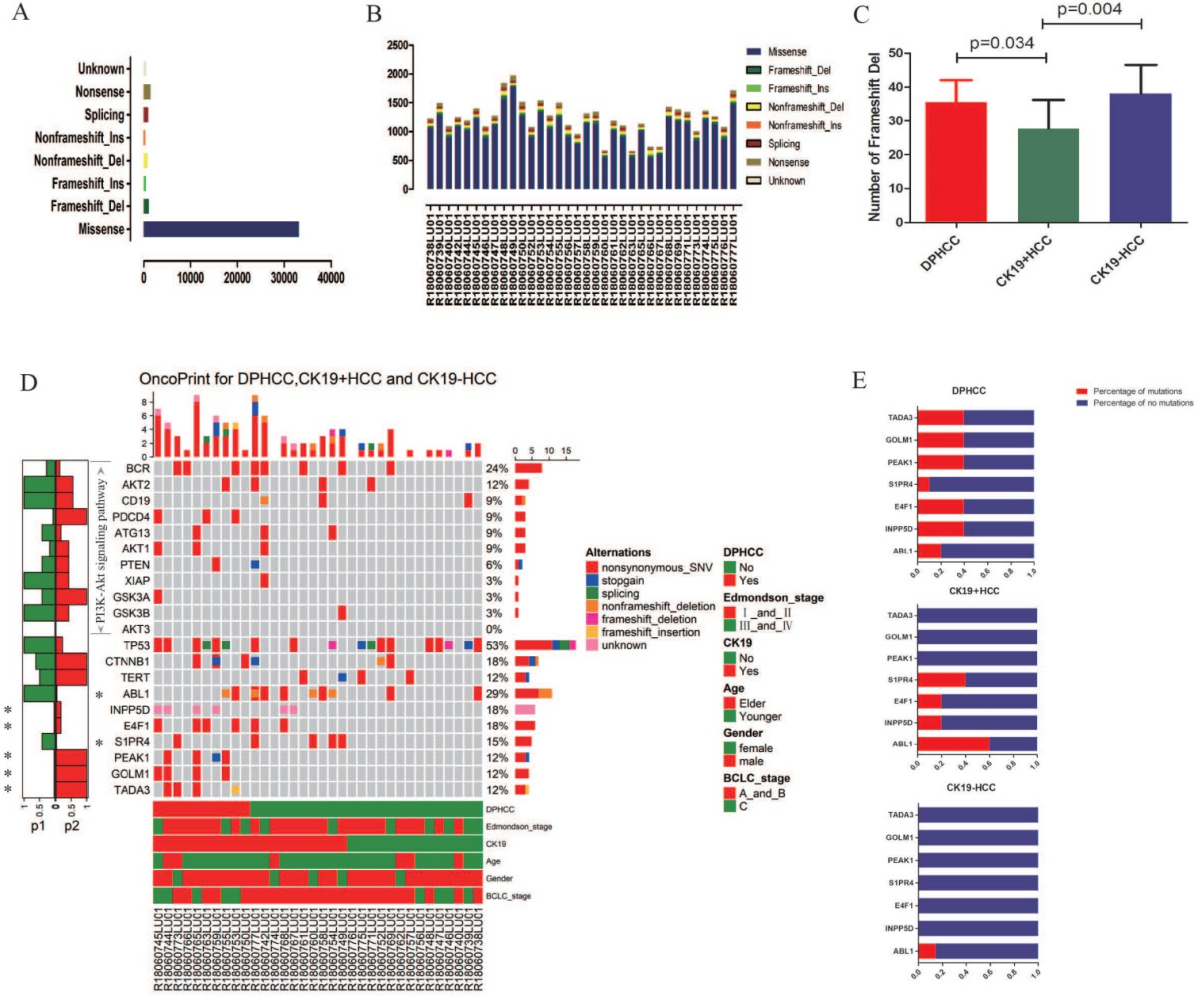
Variants identified in 34 hepatocellular carcinomas (HCC) using whole exome sequencing. (**A**) Total number of variants found in 34 HCC sample. (**B**) Number of each type of variant identified in each sample. (**C**) Number of Frameshift Del in DPHCC, CK19+HCC, and CK19-HCC. (**D**) Mutational landscape and the clinical information of 34 hepatocellular carcinomas. The lower side of Figure 2D shows the details of tumor mutation status and the clinical information of each patients. The middle panel of Figure 2D shows the genetic alterations type. The right barplot shows the mutational frequency of each gene. The left barplot emphasizes the significant degree of mutation status of each gene, and the p values (p1 and p2 represent P values for DPHCC versus CK19-HCC and CK19+HCC versus CK19-HCC, respectively). (**E**) Mutation rates of seven differentially mutated genes in DPHCC, CK19+HCC and CK19-HCC.

WES was performed in 34 patients with HCC. In 34 patients with hepatocellular carcinoma, we detected missense mutations at 33,136 sites, nonsense mutations at 1470 sites, frameshift del mutations at 1083 sites, splicing mutations at 1019 sites, non-frameshift del mutations at 905 sites, unknow mutations at 544 sites, frameshift ins mutations at 479 sites, and non-frameshift ins mutations at 382 sites (Figure 2A). In 10 DPHCC, 10 CK19+HCC, and 14 CK19-HCC, missense was the most common type of site mutation, followed by nonsense (Figure 2B). Among the eight mutation types, the number of mutation sites in frameshift del was significantly higher in DPHCC and CK19-HCC than in CK19+HCC (Figure 2C).

We identified seven genes with different mutation frequency between 10 DPHCC, 10 CK19+HCC, and 14 CK19-HCC, as well as commonly known hepatocellular carcinoma driver genes (TP53, TRET, CTNNB1) and related molecules involved in the PI3K-Akt signaling pathway. In 34 patients with hepatocellular carcinoma, the mutation rate of TP53 was as high as 52.94%, and that of CTNNB1 and TERT was 17.65% and 11.76%, respectively (Figure 2D). The other seven differentially mutated genes, including ABL1 (DPHCC20%, CK19+HCC60% and CK19-HCC14.29%), INPP5D (DPHCC40%, CK19+HCC20% and CK19-HCC0%), E4F1 (DPHCC40%, CK19+HCC20% and CK19-HCC0%), S1PR4 (DPHCC10%, CK19+HCC40% and CK19-HCC0%), PEAK1 (DPHCC40%, CK19+HCC0% and CK19-HCC0%), GOLM1 (DPHCC40%, CK19+HCC0% and CK19-HCC0%), and TADA3 (DPHCC40%, CK19+HCC0% and CK19-HCC0%) (Figure 2E). Interestingly, we found that almost all of these differentially mutated genes were highly mutated in DPHCC or CK19+HCC, whereas the mutation rate in CK19-HCC was mostly 0. More surprisingly, most of these highly mutated genes in DPHCC and CK19+HCC are related to the development of cancer, immunity, and even the progression of liver disease. Among them, ABL1 is a protooncogene that encodes a protein tyrosine kinase involved in a variety of cellular processes 68, 69. E4F1 may act as an ubiquitin ligase to mediate the ubiquitination of chromatin-associated TP53 70, 71. The protein encoded by the PEAK1 may play a role in the regulation of cell proliferation and cancer metastasis 72-74. TADA3 is involved in stabilizing and activating p53 tumor suppressor protein 75, 76. Mutations in INPP5D are associated with defects in the immune system and cancer. S1PR4 may be involved in immune cell migration processes 77. GOLM1, moreover, has been reported to be associated with the development of liver diseases. With the aggravation of liver injury, GOLM1 expression level showed a significant up-regulation 78.

To further explore whether the expression of these differentially mutated genes in DPHCC was abnormal, we examined their expression. Unfortunately, the expression of these mutated genes was not significantly abnormal in our 34 samples. Subsequently, we downloaded 374 liver cancer samples from the TCGA database. 374 cases of HCC were divided into high expression group (top 15% of expression) and low expression group (85% lower expression) according to the expression level of gene KRT19 (target gene of CK19). To our surprise, GOLM1 was significantly overexpressed in the CK19 high expression group. It has been reported that high expression of GOLM1 is associated with worsening of liver cancer and poor prognosis 79, 80.

**Figure 3:**
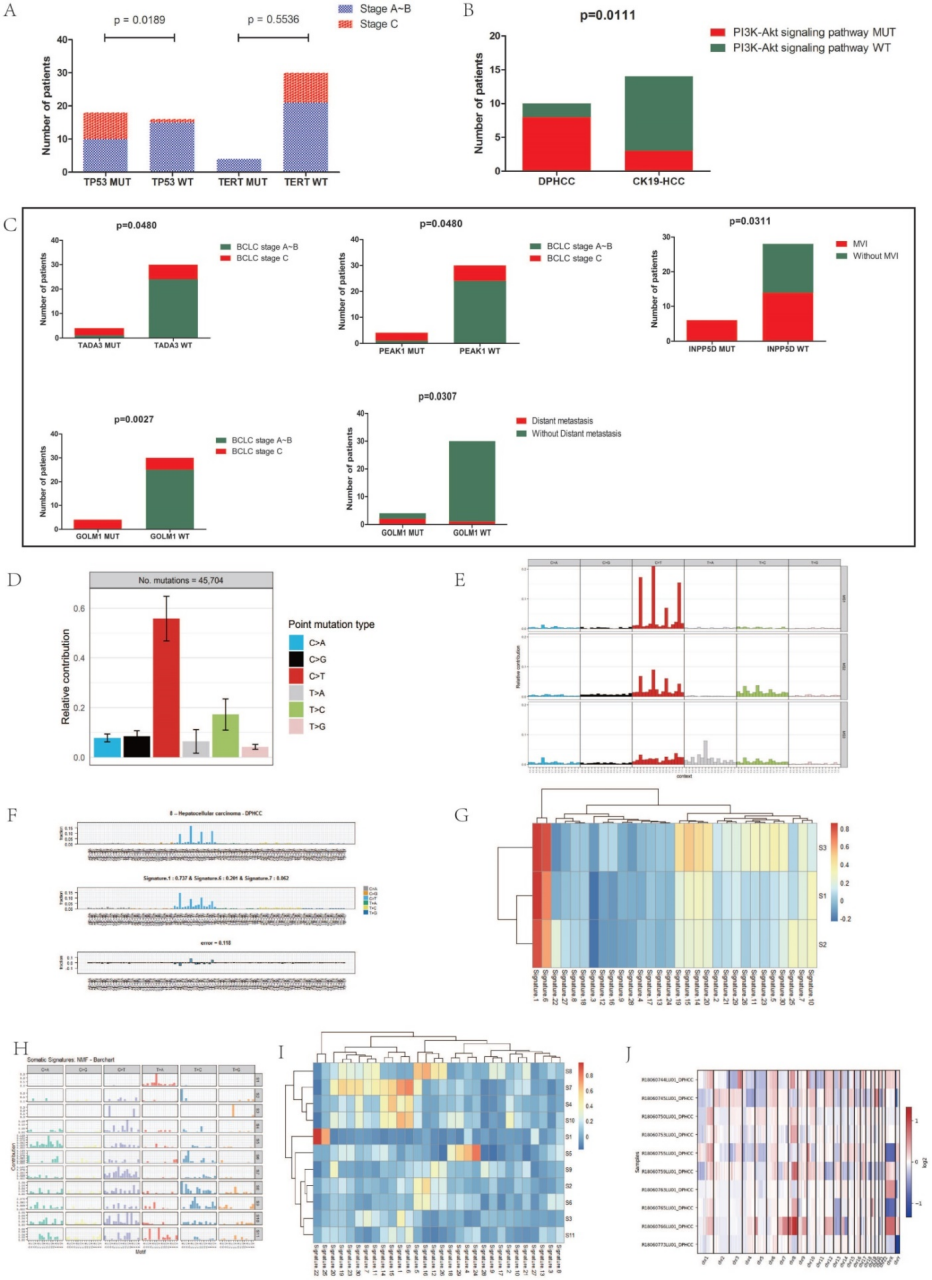
(**A**) Associations of BCLC stage with TP53 and TERT gene mutations. (**B**) Association of PI3K-Akt signaling pathway mutation with DPHCC and CK19-HCC. (**C**) Association of TADA3, PEAK1, INPP5D and GOLM1 gene mutations with clinical parameters. (**D**) The frequency of six substitution patterns in DPHCC. (**E**) Mutational signatures in DPHCC identified using the R package “MutationalPatterns” (using NMF to identify three signatures). NMF: Nonnegative Matrix Factorization. (**F**) Mutational signatures in DPHCC identified using the R package “deconstructSigs”. (**G**) Correlation between mutational signatures of DPHCC identified using the R package "SomaticSignatures" and mutational signatures of COSMIC. (**H**) Mutational signatures in 154 Asian patients with hepatocellular carcinoma. (**I**) Correlation between mutational signatures of 154 Asian patients with hepatocellular carcinoma and mutational signatures of COSMIC. (**J**) The heatmap of somatic CNVs for 10 DPHCC samples.

We further analyzed the correlation between gene mutations and tumor progression. TERT promoter mutations occur in the early stage of hepatocellular carcinoma, while TP53 changes occur in the late stage of invasive tumors 26. Analysis of the relationship between mutations in TP53 and TERT genes and cancer stage in 34 patients with hepatocellular carcinoma revealed that TP53 mutations were significantly up-regulated in the advanced stage of hepatocellular carcinoma (P=0.019), while TERT gene mutations were not significantly altered in the early stage of hepatocellular carcinoma (P=0.6) (Figure 3A).

In addition to mutations in cancer-driving genes, activation of signaling pathways may also lead to cancer. The occurrence of HCC is associated with the activation of many signaling pathways, such as AKT signaling pathway, Wnt signaling pathway, and PI3K/AKT signaling pathway, etc. We found that compared with CK19-HCC, PI3K-Akt signal pathway (hsa04151) is more likely to be altered in DPHCC (80% for DPHCC, 21.4% for CK19-HCC, P = 0.011) (Figure 3B).

Next, we conducted a correlation analysis between HCC differentially mutated genes and clinical characteristics. We found that TADA3 and PEAK1 mutations were significantly associated with advanced tumor stage (P = 0.048 and P = 0.048), INPP5D mutations were significantly associated with microvascular invasion (MVI) (P = 0.031), and GOLM1 mutations were significantly associated with advanced tumor stage and Distant metastasis (P = 0.003 and P = 0.031, respectively) (Figure 3C).

### Mutational patterns in DPHCC

To explore the specific etiological factors that may contribute to the mutagenesis of DPHCC, we performed mutational spectrum analysis of DPHCC to categorize their mutational signature and to identify functional mutagenic processes in DPHCC. In DPHCC, the C > T transversion was the most frequent of six substitution patterns (C > A, C > G, C >T, T > A, T > C, and T > G) (Figure 3D), which is similar to the results of previous studies on HCC 81. Compared with the traditional HCC with a far more heterogeneous composition of individual mutational COSMIC signatures studied by previous researchers 81, the COSMIC signature of DPHCC shows that mutation signature 1 is the major signature affecting DPHCC patients (Figure 3E).

The results of two R packages: “deconstructSigs” and “SomaticSignatures” were similar to those of “MutationalPatterns”. The results of the “deconstructSigs” package analysis showed that C > T was the most common SNV. The highest contribution was signature 1, accounting for 73.7%. Signatures 6 and 7 accounted for 20.1% and 6.2%, respectively (Figure 3F).

Similar to the results of the first two packages, we used the “SomaticSignatures” package to decompose the mutations of DPHCC into three mutational signatures (S1, S2 and S3). S1, S2 and S3 all had higher C > T mutation frequencies, which corresponded to Catalog of Somatic Mutations in Cancer (COSMIC; https://cancer.sanger.ac.uk/cosmic/signatures/) Signature 1 (Figure 3G).

We decomposed the mutations of 154 Asian patients with hepatocellular carcinoma downloaded from TCGA into 11 mutational signatures (Figure 3H). Except for S1, S5 and S7, none of the other signatures corresponded to mutation signatures in the COSMIC database (Figure 3I). Comparing with the COSMIC signatures, we found that S1 was associated with aristolochic acid exposure, S5 with aflatoxin exposure, and S7 with DNA mismatch repair deficiency (dMMR).

Signature 1 is one of the mutation signatures associated with the endogenous mutation process. Because stem cells of different cancer types divide at different rates, signature 1 acts like a clock and the rates of acquisition of signature 1 mutations differ markedly over time. Signature 1 may therefore be a cell division/mitosis clock 82-84. Signature 6 is associated with defective DNA mismatch repair and is found in microsatellite unstable tumors. In addition, signature 6 is associated with large numbers of small insertions and deletions (ID) 1 and 2 mutations, which are characterised respectively by small (usually 1 bp) insertions and deletions of T at mononucleotide T repeats.

### Copy number variants profiles

We identified a total of 381 CNVs from the 10 DPHCC samples with a mean of 38, of which 205 were CN-gains and 176 were CN-losses (Figure 3J). The results showed that the heterogeneity of copy number variation was relatively large in DPHCC. Among the 10 DPHCCs, chromosome 11 was relatively stable, and the chromosome 1q and 8q mostly showed CN-gains (Figure 3J, Figure S1). In the chromosome 1q, Tumor Potentiating Region (TPR) and Odorant Response Abnormal 4 (ODR4) have a higher frequency of CN-gain.

### Identification of DEGs

**Figure 4:**
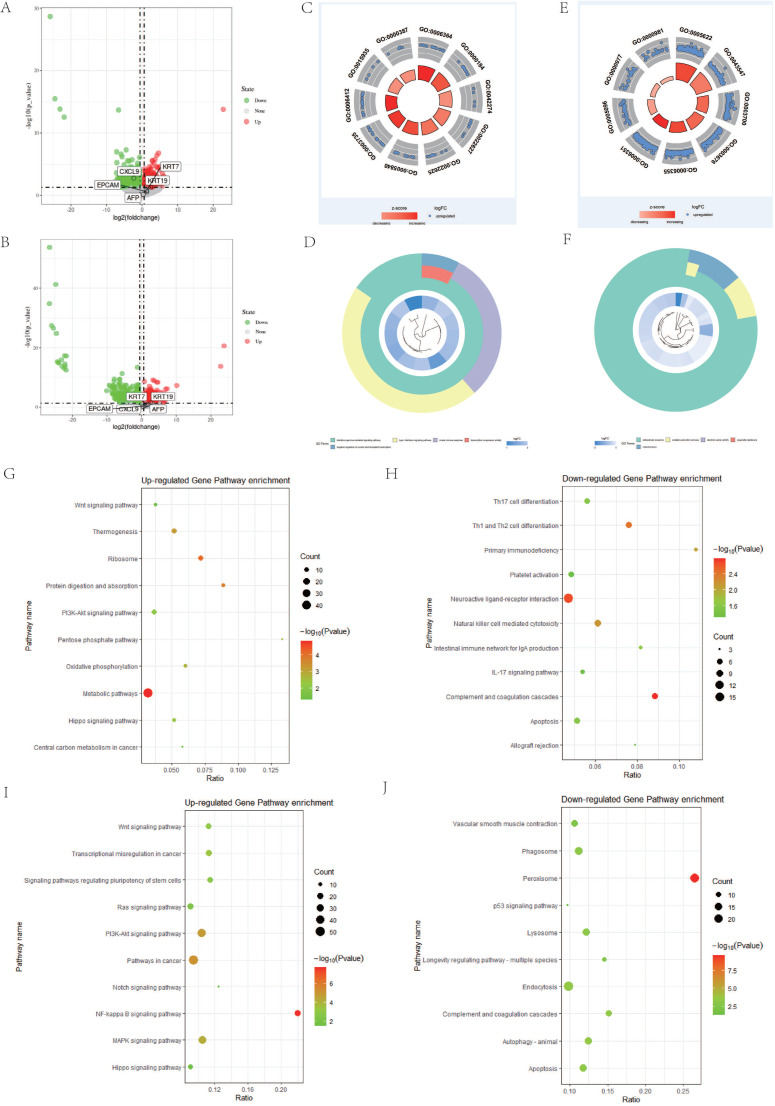
Differential gene and pathway enrichment analysis. (**A and B**) are the differential genes volcano plot of DPHCC and CK19+HCC, respectively. (**C and D**) GO pathway analysis of differentially up-regulated and down-regulated genes between DPHCC and CK19-HCC, respectively. (**E and F**) GO pathway analysis of differentially up-regulated and down-regulated genes between CK19+HCC and CK19-HCC, respectively. (**G and H**) KEGG pathway analysis of differentially up-regulated and down-regulated genes between DPHCC and CK19-HCC, respectively. (**I and J**) KEGG pathway analysis of differentially up-regulated and down-regulated genes between CK19+HCC and CK19-HCC, respectively.

To further investigate whether there are other abnormally expressed genes that affect the poor prognosis of DPHCC, we performed differential expression analysis. In HCC, CK19-HCC patients are the group with relatively good prognosis, which is defined as the control group. However, the prognosis of DPHCC and CK19+HCC was a relatively poor 8 and were treated as independent experimental groups. Based on P-value < 0.05 and | Fold Change (FC)| > 1.5, a total of 1410 DEGs were identified, including 624 up-regulated genes and 786 down-regulated genes in the DPHCC (Figure 4A). There were 3464 genes that were dysregulated in CK19+HCC, of which 1807 were up-regulated genes and 1657 were down-regulated (Figure 4B). As shown in Figure 4A and B, compared with CK19-HCC, KRT19 expression showed a significant up-regulation in DPHCC and CK19+HCC. Other stem cell markers, such as KRT7, EPCAM and AFP, also showed a trend to be up-regulated, but there was no statistical difference.

### Gene set enrichment analysis

To further understand the role of the differentially expressed genes in DPHCC and CK19+HCC, we used DAVID and KOBAS to analyze the enrichment of GO and KEGG pathways, respectively. The enrichment analysis of up-regulated genes and down-regulated genes was carried out separately.

There are many interesting enrichment pathways for GO enrichment. The differentially up-regulated genes of DPHCC are mainly enriched in transcription related pathways, such as rRNA processing (GO: 0006364), ribosomal small subunit biogenesis (GO: 0042274), translation (GO: 0006412), etc. (Figure 4C). However, the down-regulated genes are mainly enriched in immune and transcription related pathways, such as type I interferon signaling pathway (GO: 0060337), innate immune response (GO: 0045087), negative regulation of nucleic acid-templated transcription (GO: 1903507), etc. (Figure 4D). Similarly, differentially up-regulated genes in CK19+HCC are also mainly enriched in pathways related to transcription and translation, such as transcription factor activity, sequence-specific DNA binding (GO: 0003700), regulation of transcription, DNA-templated (GO: 0006355), transcription, DNA-templated (GO: 0006351), etc. (Figure 4E). And the differentially down-regulated genes were mainly enriched in oxidation-reduction process (GO: 0055114) and electron carrier activity (GO: 0009055) (Figure 4F).

For the enrichment of KEGG, we found many important pathways related to cancer. The differentially up-regulated genes of DPHCC are mainly enriched in cancer-related pathways, such as PI3K-Akt signaling pathway (hsa04151) and Wnt signaling pathway (hsa04310) (Figure 4G). Interestingly, there are reports that molecular mutations or copy number variations involved in the PI3K-Akt signaling pathway may be associated with higher PI3K-Akt signaling 85. Similarly, our results also showed that the PI3K-Akt signaling pathway had a significantly higher mutation frequency in DPHCC (Figure 3B), and the differentially up-regulated genes in DPHCC were enriched in the PI3K-Akt signaling pathway (Figure 4G). Differentially down-regulated genes were mainly enriched in immune-related pathways, such as Complement and coagulation cascades (hsa04610), Th1, and Th2 cell differentiation (hsa04658), Natural killer cell mediated cytotoxicity (hsa04650) and IL-17 signaling pathway (hsa04657) (Figure 4H). Surprisingly, CK19+HCC differentially up-regulated genes were also mainly enriched in cancer-related pathways, such as NF-kappa B signaling pathway (hsa04064), PI3K-Akt signaling pathway (hsa04151), Wnt signaling pathway (hsa04310), etc. (Figure 4I). The differentially down-regulated genes were mainly enriched in peroxisome (hsa04146), autophagy - animal (hsa04140), and lysosome (hsa04142) (Figure 4J).

### PPI network analysis

**Figure 5:**
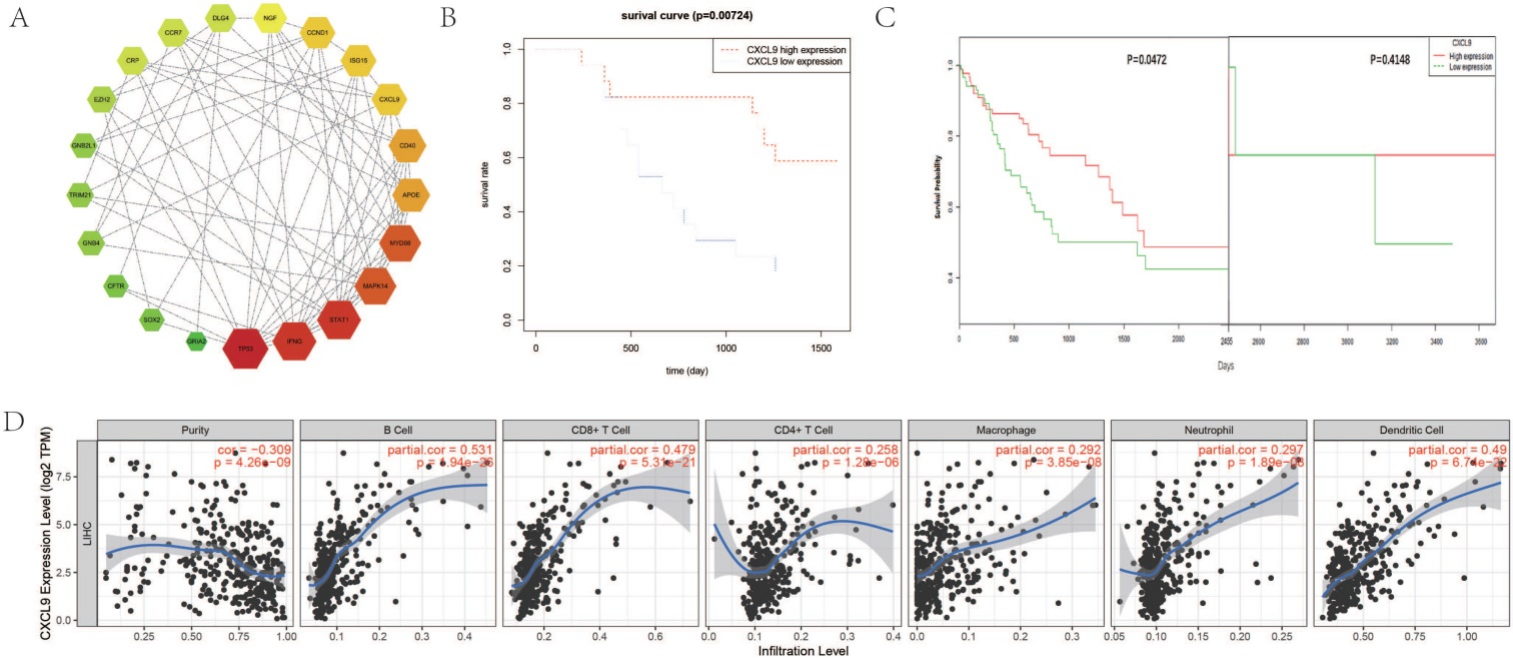
Survival analysis of CXCL9 and its correlation with immune cell level and tumor immune infiltrating cells in HCC. (**A**) The interactions and protein-protein networks of the top 21 hub genes. (**B**) Analysis of overall survival of CXCL9 in 34 HCC patients. (**C**) Analysis of CXCL9 overall survival in HCC patients from TCGA database. (**D**) Correlation of gene expression with tumor purity and immune invasion.

We performed PPI network analysis of differentially expressed genes in DPHCC to identify key genes and related gene modules involved in interactions as well as affecting DPHCC. The results of STRING analysis showed that the PPI network of DEGs was constructed, consisting of 1158 nodes and 3715 edges, with an average node degree of 6.42 and the PPI enrichment p-value < 1.31 × 10^-9^. The degree of a node (protein) in a network (interactome) is the number of links (interactions) to other nodes, or simply the number of contacts 86. Based on the results of STRING analysis, the cytoHubba plugin in Cytoscape reported 207 hub genes of DPHCC with the criterion of degree 11 connected nodes. Hub genes are highly connected genes in the network, which are expected to play an important role in understanding the biological mechanism of response under stress/conditions 87. Subsequently, the top 22 hub genes with the criterion of degree ≥30 connected nodes were again submitted to STRING to verify the interaction among them. The PPI network consisted of 21 nodes and 75 edges, with a mean node degree of 6.82, and showed a closer protein interaction among the hub genes (Figure 5A). Then, hub gene CXCL9 88, which is related to the chemotaxis of activated T cells and has a higher degree value (degree=8), was selected as a candidate gene for further analysis.

### Survival analysis of CXCL9 and its correlation with immune infiltration level

We performed a survival analysis on CXCL9 and validated it with OncoLnc online tool. Our results showed that low expression of CXCL9 had a worse prognosis (Figure 5B) and OncoLnc online tool analysis of HCC survival data in TCGA also showed that low expression of CXCL9 had a worse prognosis. We corrected the bias inherent in the analysis of time-to-event outcomes between groups identified during study follow-up with landmark analyses. After correction, the results remained consistent with ours, and the survival rate of patients with low CXCL9 expression decreased significantly over five years (P=0.0472) (Figure 5C). Subsequently, we analyzed the correlation between the expression of CXCL9 and the level of immune infiltration. Scatterplots first show gene expression levels for tumor purity. The tumor microenvironment is a complex non-tumor cell environment composed mainly of immune cells around the tumor cells. Genes highly expressed in cells in the microenvironment are expected to have negative associations with tumor purity, while the opposite is expected for genes highly expressed in the tumor cells 65. As shown in Figure 5D, CXCL9 expression was negatively correlated with tumor purity (P=4.26 × 10^-9^), but positively correlated with B cell level (P=1.94 × 10^-26^), CD8+T cell level (P=5.31 × 10^-21^), CD4+T cell level (P=1.28 × 10^-6^), neutrophil level (P=1.89 × 10^-8^), macrophage level (P=3.85 × 10^-8^), and dendritic cell level (P=6.74 × 10^-22^).

### Identification of prognostic risk factors and prognostic value assessment

**Figure 6:**
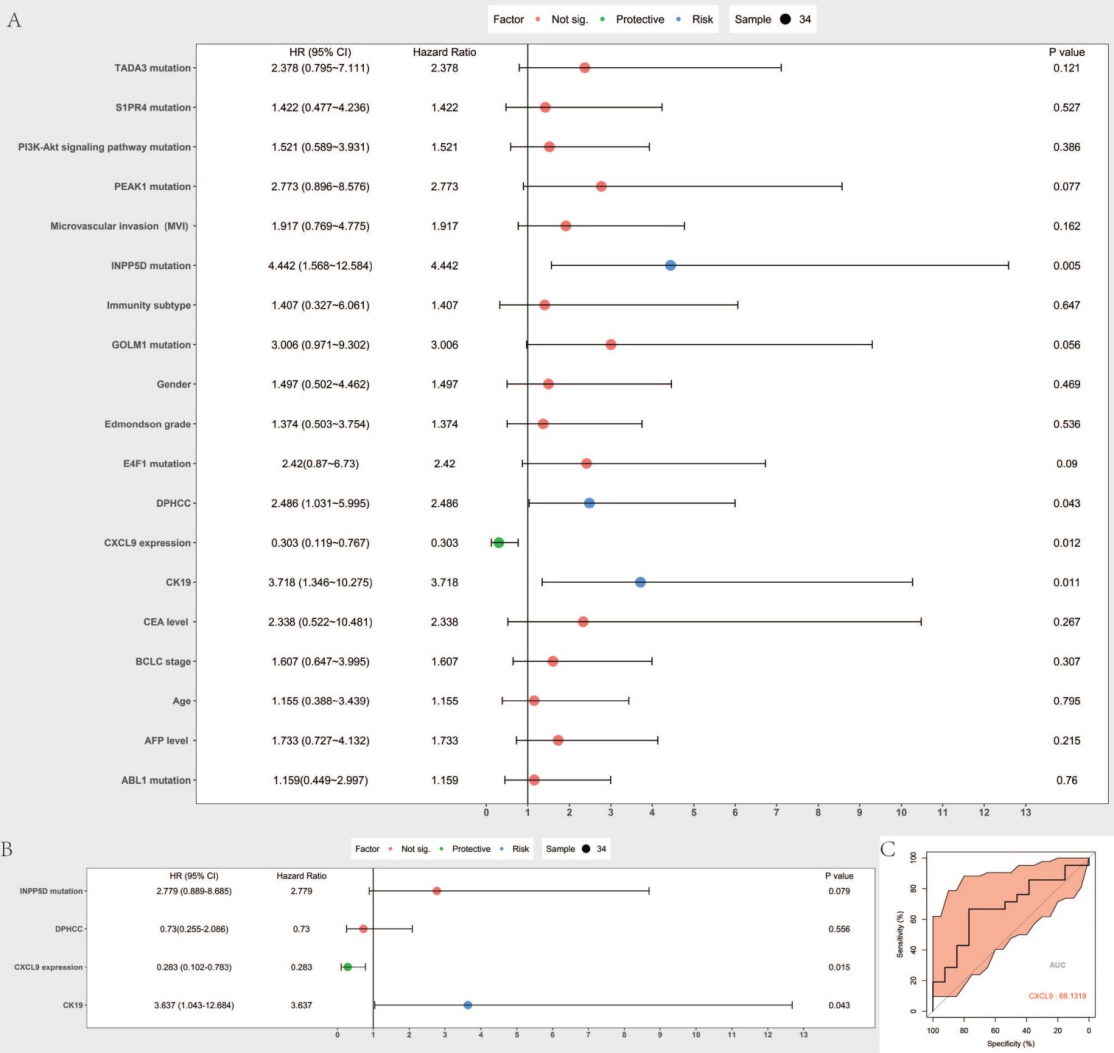
Prognostic value assessment of CXCL9. (**A and B**) are Forest map of univariate and multivariate Cox regression analysis, respectively. The line shows 95% CI, and the position of the square on the line represents the odds ratio. (**C**) Receiver operating characteristic curve of CXCL9. ROC was performed for CXCL9 for the prognostic value in HCC.

Cox proportional hazard model was established to predict prognostic risk factors. As Figure 6A reveals, univariate analysis showed the overall survival was significantly correlated with DPHCC (P=0.043), CK19 (P= 0.011), CXCL9 expression (0.012), INPP5D mutation (P= 0.005). Multivariate analysis showed that CK19 (P= 0.043) and CXCL9 expression (P= 0.015) were independent risk factors in predicting the prognosis of hepatocellular carcinoma patients (Figure 6B). We further validated CXCL9 expression with online web tools OncoLnc and TIMER for COX regression. Both results showed that CXCL9 expressions were independent risk factors in predicting the prognosis of liver cancer patients (Figure S2A, B). Furthermore, the prognostic value, including sensitivity and specificity of CXCL9, was performed by ROC analysis. In the receiver operating characteristic (ROC) curve, the area under the curve (AUC) is 0.681, demonstrating that CXCL9 assessment is accurate (sensitivity: 0.666, specificity: 0.769) (Figure 6C).

## Discussion

The World Health Organization classifies primary liver cancer into either HCC, ICC, or CHC 89, and there are few reports on DPHCC. DPHCC is more aggressive and malignant than HCC because it expresses both markers of HCC and cholangiocarcinoma 7. The prognosis of DPHCC patients is also significantly worse than that of HCC patients 90.

Compared to genome wide association studies, WES compares genomes with base-pair accuracy and reveals rare genetic variations 91. Here, WES and RNA sequencing were used to elucidate the molecular mechanisms underlying DPHCC pathogenesis.

DPHCC has a unique gene mutation profile, and several genes with differential mutations in DPHCC, CK19+HCC, and CK19-HCC were identified, including ABL1, E4F1, PEAK1, TADA3, INPP5D, S1PR4, and GOLM1.

ABL1 is a protooncogene that encodes a protein tyrosine kinase involved in a variety of cellular processes 68, 69. E4F1 is a transcription factor in the Gli-Kruppel family that was identified as a cellular target of the adenoviral oncoprotein E1A 92. It is a multifunctional protein with transcriptional and atypical ubiquitin E3 ligase activities that plays a role in cell survival and proliferation 70, 71. PEAK1 is involved in the regulation of cell migration, proliferation, and cancer metastasis 72-74, 93, and TADA3 is involved in stabilizing and activating p53 and plays a role in the cellular response to DNA damage 75, 76. Mutations in INPP5D are associated with defects in the immune system and cancer 94. S1PR4 may be involved in specific cell migration processes 77. GOLM1 is associated with the development of liver disease and serves as a marker of liver injury 78, and has been suggested as a potential serum marker for the diagnosis of HCC 95.

While GOLM1 is up-regulated in the CK19 high expression group in TCGA database, it showed no abnormal expression in our samples. This may be due to an insufficient sample size. In addition, our HCC sample subtypes are not identical to the 374 HCC sample subtypes from TCGA, and our sample subtypes are even rarer. All our 34 samples were hepatocellular carcinoma samples, which were subdivided into three subtypes: CK19-HCC, CK19+HCC and DPHCC according to the markers expressed by hepatocellular tumors by immunohistochemistry. However, even if the expression of gene KRT19 (target gene of CK19) can be measured by RNA-seq in 374 liver cancer samples from TCGA database to classify liver cancer into CK19-HCC and CK19+HCC, DPHCC cannot be subdivided further. Because according to the case diagnosis criteria, DPHCC not only expresses one of the markers of hepatocellular carcinoma and one of the markers of cholangiocarcinoma, but also the two markers are co-expressed and the proportion of tumor cells with co-expression is more than 15%. The TCGA database of liver cancer samples can evaluate the expression of markers based on gene expression, but cannot evaluate whether they co-express the two markers and the proportion of tumor cells with co-expression.

Most of the mutated genes showed no abnormal expression, which may be due to the limited sample size, and the relationship between gene mutation and expression could not be accurately reflected in a small cohort. More data and larger cohorts are needed to accurately explore which gene mutations cause abnormal expression and ultimately worsen tumor progression.

There are a large number of high-frequency mutations in key genes in DPHCC, and whether their abnormal expression leads to a worse prognosis remains to be elucidated.

In our study, the mutation rate of TERT was only 11.76%, which is inconsistent with the established high mutation rate in liver cancer. The low rate of TERT mutation may be due to poor coverage of the TERT promoter region by WES 96. In another of our research projects, TERT promoter mutations were targeted in 136 patients with HCC, and the mutation rate was as high as 66.9% (91/136).

We found a significant increase in the frequency of mutations in the PI3K-Akt signaling pathway in DPHCC samples. Similar studies have reported that 63% of the cases had at least one somatic mutation or copy number variation involving the PI3K-Akt signaling pathway in tumor samples, and biomolecular mutation or copy number variation in this pathway may be associated with increased PI3K-Akt signaling 85.

After the WES of 34 HCC patients, we found that DPHCC patients exhibited a higher frequency of gene and pathway mutations than CK19-HCC patients, indicating that abnormal PI3K-Akt signaling may be an inherent characteristic of DPHCC.

Upon analysis of the mutational signatures in DPHCC through signature enrichment analysis, we found that the Signature 1 pattern was increased in DPHCC. Signature 6 pattern also accounts for a small proportion in DPHCC. Moreover, we analyzed with three different R packages, and the results were roughly the same, indicating that the results of the analysis were reliable. Compared with the mutation signatures of hepatocellular carcinoma patients studied previously 81 and the mutation signatures of 154 Asian hepatocellular carcinoma patients in TCGA database, the mutation signatures composition of DPHCC patients is relatively simple.

CNVkit is a command-line toolkit and Python library available for Ubuntu or Debian Linux and Mac OS X. However, an important issue is that to use this tool the user must first install several Python and R packages, and CNV calling requires the use of multiple command lines in a specific order, meaning that users must have moderate programming skills. In this study, we conducted WES to explore the relationship between CNVs in two subtypes of hepatocellular carcinoma, CK19-HCC and DPHCC. The results showed that the heterogeneity of copy number variation was relatively large in DPHCC. Compared with CK19-HCC, chromosome 11 of DPHCC is relatively stable, and the chromosome 1q and 8q mostly showed CN-gains. The frequent trend of CN-gain of TPR and ODR4 in chromosome 1q may indicate that CN-gain of TPR and ODR4 plays a role in the progression of DPHCC, while chromosome 11 has little effect on the transformation of this hepatocellular carcinoma subtype. However, due to the limited sample size, copy number variations leading to subtype changes in hepatocellular carcinoma still need to be validated in larger cohorts.

RNA sequencing followed by differential expression analysis and enrichment analysis were applied to investigate the abnormity of DPHCC in gene expression level.

We found 1410 dysregulated genes in DPHCC samples, of which 624 were up-regulated and 786 were down-regulated. In CK19+HCC samples, 3464 genes were dysregulated, with 1807 up-regulated and 1657 down-regulated. CXCL9 expression was significantly down-regulated in DPHCC, but not in CK19+HCC. In our cohort, the expression of several stem cell markers examined tended to be up-regulated in both DPHCC and CK19+HCC, but none of them were significantly statistically different, except for KRT19.

GO enrichment results showed that the up-regulated genes of DPHCC and CK19+HCC samples were abundantly enriched in pathways related to ribosome generation, transcription up-regulation, and activation of transcription factors. The acceleration of ribosome biogenesis and transcription is particularly important for rapid cell proliferation 97, 98, which may accelerate tumor cell growth. The down-regulated genes in DPHCC samples were abundantly enriched in immune-related pathways. The body's immune response to tumors depends on the balance between the antigenicity of tumors and the microenvironment of tumor tissues 99, and the down-regulation of immune-related pathways is conducive to the establishment of an immunosuppressive environment. In this immunosuppressive environment, tumors often show poor prognosis 100.

In KEGG pathway enrichment, we also found that the up-regulated genes of DPHCC and CK19+HCC samples were enriched in many cancer-related pathways, such as PI3K-Akt signaling pathway, Wnt signaling pathway, and the NF-kappa B signaling pathway. The pathways enriched in the down-regulated DPHCC genes were mainly immune-related. The PI3K-Akt signaling pathway plays an important role in regulating the normal growth, metabolism, and survival of cells. Inhibition of PI3K-Akt signaling can inhibit cell proliferation, attenuate the proliferation ability of hepatoma cells, and reduce the invasiveness of tumors 101-103. Conversely, activation of the PI3K-Akt signaling pathway can promote the growth, metastasis, and progression of HCC 104-107. It has been reported that molecular mutations or copy number variations involved in the PI3K-Akt signaling pathway may be associated with higher PI3K-Akt signaling 85. Here, the WES results revealed that the mutation rate of the PI3K-Akt pathway in DPHCC tissues is significantly higher than that in CK19-HCC tissues. This may contribute to DPHCC having a worse prognosis than CK19-HCC. The Wnt pathway is a key component of embryonic development and tissue homeostasis, which is associated with cell survival, proliferation, migration, and invasion 108. Activation of Wnt signaling promotes the occurrence and development of liver cancer, as well as self-renewal of liver CSCs and tumor invasion and migration 109-113. Up-regulated Wnt signaling predicts a worse prognosis in HCC patients.

The GO and KEGG analyses revealed that both DPHCC and CK19+HCC are more aggressive and have a worse prognosis than CK19-HCC. Moreover, the immune environment of DPHCC may be suppressed and the prognosis may be worse than CK19+HCC.

To resolve the interactions between DEGs in DPHCC, a close interacting PPI network was found, which included 21 genes, namely TP53, IFNG, STAT1, MAPK14, MYD88, APOE, CD40, CXCL9, ISG15, CCND1, NGF, DLG4, CCR7, CRP, EZH2, GNB2L1, TRIM21, GNB4, CFTR, SOX2, and GRIA2. Given the higher degree of CXCL9 and its association with immunity, CXCL9 was selected for further validation in TCGA cohort and our patient samples. Results showed that the expression of CXCL9 was negatively correlated to tumor purity, and positively correlated to the levels of immune cells, including B cells, CD8+T cells, CD4+T cells, macrophages, neutrophils, and dendritic cell. Similarly, TCGA database analysis showed that low CXCL9 levels were associated with poor overall survival.

To exclude the impact of other interfering factors on survival prognosis, we conducted a univariate and multivariate analysis of Cox proportional risk regression on risk factors that may affect survival.

While multivariate analysis of Cox proportional risk regression showed that genes and pathways with high-frequency mutations in DPHCC could not be independent risk factors, the results demonstrated that DPHCC is a new HCC subtype with extremely unstable gene mutation status, and is accompanied by an immunosuppressive environment. As a high-frequency of mutated genes and pathways cannot be independent risk factors, it may be that DPHCC pathogenesis is not determined by a single gene mutation, but by mutations in multiple genes and pathways, together with suppression of the immune system.

The results of Cox proportional risk regression further confirmed that low expression of CXCL9 was not conducive to patient survival. CXCL9 is an independent risk factor in predicting the prognosis of HCC patients.

Chemokines are a class of polypeptides that contain 4 conserved cysteine residues and have chemotactic functions 114. Chemokines play important roles in embryonic development, angiogenesis, hematopoiesis, atherosclerosis, cancer and other pathophysiological processes. CXC chemokine-mediated regulation of angiogenesis has been shown to play a key role in solid tumors. ELR-containing CXC chemokines can promote angiogenesis and thus promote tumor growth, whereas CXCL9, which is mainly induced by IFN - γ and synthesized in lymphocytes, monocytes, and fibroblasts, as a non-ELR-containing CXC chemokines, can inhibit angiogenesis and thus play a cancer suppressive role.

CXCL9 is produced by macrophages, endothelial cells, hepatocytes, and tumors 115. As a CXCR3 ligand, CXCL9 acts mainly as a chemoattractant to activate immune cells, including T cells and natural killer cells 116. CXCL9 stimulates lymphocytes to enter tumors and enhances anti-tumor immune monitoring. Gorbachev et al. showed that in mouse skin fibrosarcoma, CXCL9-deficient tumor cells interfered with the aggregation of immunocompetent cells, making CXCL9-deficient tumor cells more tumorigenic 117. Additionally, a high expression of CXCL9 has been associated with a good survival rate after surgery in ovarian and colorectal cancers 118. There are also studies that combine IL-2 and CXCL9 to slow angiogenesis and tumor progression 119.

Endogenous CXCL9 affects tumor progression and postoperative survival in intrahepatic cholangiocarcinoma patients by regulating tumor-infiltrating NK cells, which was also validated in mouse models 120. High expression of CLCX9 is associated with high infiltration of NK cells, while NK cells, as immune-related cells in the liver, can not only prevent cancer cells from invading the liver, but also kill cancer cells through a variety of cytotoxic pathways. CXCL9 released by tumor cells can regulate the enrichment of NK cell subset that expresses tumor necrosis factor-related apoptosis-in-ducing ligand (TRAIL+NK cell) to tumor tissues. Therefore, tumor cells with low expression of CXCL9 make insufficient enrichment of TRAIL+NK cell, which leads to tumor cell growth 121. In addition, the expression of CXCL9 was also associated with the infiltration of T cells. Highly infiltrating T cells can control tumor growth through IFN-gamma-dependent pathway, whereas high infiltration of T cells is associated with high expression of CXCL9 122. Tumors expressing CXCL9 deficiency fail to recruit cytotoxic CD8 T cells, resulting in accelerated tumor growth. High expression of CXCL9 accelerated the generation of CCL5 in tumor microenvironment, and tumors with elevated expression of CXCL9 and CCL5 showed higher immunoreactivity and immune checkpoint inhibition response 123. Many reports have confirmed that tumor-derived CXCL9 is a tumor suppressor 116, and is associated with a good prognosis and a good response to chemotherapy 124-126. Here, CXCL9 was significantly down-regulated in DPHCC tissues and not CK19+HCC tissues, and the immune microenvironment was suppressed, which may contribute to DPHCC having a worse prognosis than CK19+HCC.

The present study is limited by the small number of cases, and there is a need for a larger cohort for validation. Regardless, both our data and TCGA data showed that reduced CXCL9 expression was associated with a worse prognosis. We speculate that the presence of a severe immunosuppressive environment in DPHCC may be associated with the activation of PI3K-Akt signaling pathway mutations and the significant down-regulation of CXCL9 expression in DPHCC tissues.

In conclusion, despite the limitation of insufficient sample size, we explored the molecular mechanisms underlying DPHCC pathogenesis. These results provide useful insight into the disease mechanisms, with potentially important clinical implications.

## Supplementary Material

Supplementary figures.

Supplementary tables.

## Figures and Tables

**Table 1:** Clinicopathological data of hepatocellular carcinoma patients

Parameters	n (%)
**Age**	
>55 yr	6 (17.65)
≤55 yr	28 (82.35)
**Gender**	
Male	29 (85.29)
Female	5 (14.71)
**DPHCC**	
positive	10 (29.41)
negative	24 (70.59)
**CK19**	
positive	20 (58.82)
negative	14 (41.18)
**Liver cirrhosis**	
Yes	11 (32.35)
No	23 (67.65)
**Tumor size**	
≤5.0 cm	16 (47.06)
>5.0 cm	18 (52.94)
**Tumors number**	
<2	20 (58.82)
≥2	14 (41.18)
**Edmondson grade**	
I~II	24 (70.59)
III~IV	10 (29.41)
**BCLC stage**	
A~B	25 (73.53)
C	9 (26.47)
**Microvascular invasion (MVI)**	
Yes	20 (58.82)
No	14 (41.18)
**Distant metastasis**	
Yes	3 (8.82)
No	31 (91.18)
**AFP level**	
>400 ng/mL	17 (50.00)
≤400 ng/mL	17 (50.00)

Abbreviations: DPHCC, Dual-phenotype hepatocellular carcinoma; CK19, Cytokeratin 19; AFP, Alpha-fetoprotein.

## References

[1] Bray F, Ferlay J, Soerjomataram I, Siegel RL, Torre LA, Jemal A (2018). Global cancer statistics 2018: GLOBOCAN estimates of incidence and mortality worldwide for 36 cancers in 185 countries. CA Cancer J Clin.

[2] Liu C-Y, Chen K-F, Chen P-J (2015). Treatment of Liver Cancer. Cold Spring Harb Perspect Med.

[3] Altekruse SF, Devesa SS, Dickie LA, McGlynn KA, Kleiner DE (2011). Histological classification of liver and intrahepatic bile duct cancers in SEER registries. Journal of registry management.

[4] Allen RA, Lisa JR (1949). Combined liver cell and bile duct carcinoma. Am J Pathol.

[5] Zhang F, Chen XP, Zhang W, Dong HH, Xiang S, Zhang WG (2008). Combined hepatocellular cholangiocarcinoma originating from hepatic progenitor cells: immunohistochemical and double-fluorescence immunostaining evidence. Histopathology.

[6] Koh KC, Lee H, Choi MS, Lee JH, Paik SW, Yoo BC (2005). Clinicopathologic features and prognosis of combined hepatocellular cholangiocarcinoma. Am J Surg.

[7] Lu XY, Xi T, Lau WY, Dong H, Zhu Z, Shen F (2011). Hepatocellular carcinoma expressing cholangiocyte phenotype is a novel subtype with highly aggressive behavior. Annals of surgical oncology.

[8] Zhang J, Qi YP, Ma N, Lu F, Gong WF, Chen B (2020). Overexpression of Epcam and CD133 Correlates with Poor Prognosis in Dual-phenotype Hepatocellular Carcinoma. J Cancer.

[9] Jung DH, Hwang S, Kim KH, Hong SM, Lee YJ, Ahn CS (2017). Clinicopathological Features and Post-resection Prognosis of Double Primary Hepatocellular Carcinoma and Intrahepatic Cholangiocarcinoma. World J Surg.

[10] Mishra L, Banker T, Murray J, Byers S, Thenappan A, He AR (2009). Liver stem cells and hepatocellular carcinoma. Hepatology.

[11] Chiba T, Kamiya A, Yokosuka O, Iwama A (2009). Cancer stem cells in hepatocellular carcinoma: Recent progress and perspective. Cancer Lett.

[12] Reya T, Morrison SJ, Clarke MF, Weissman IL (2001). Stem cells, cancer, and cancer stem cells. Nature.

[13] Sung JJ, Noh SJ, Bae JS, Park HS, Jang KY, Chung MJ (2016). Immunohistochemical Expression and Clinical Significance of Suggested Stem Cell Markers in Hepatocellular Carcinoma. J Pathol Transl Med.

[14] Kim H, Park YN (2014). Hepatocellular carcinomas expressing 'stemness'-related markers: clinicopathological characteristics. Digestive diseases (Basel, Switzerland).

[15] Takano M, Shimada K, Fujii T, Morita K, Takeda M, Nakajima Y (2016). Keratin 19 as a key molecule in progression of human hepatocellular carcinomas through invasion and angiogenesis. BMC cancer.

[16] Yoo JE, Kim YJ, Rhee H, Kim H, Ahn EY, Choi JS (2017). Progressive Enrichment of Stemness Features and Tumor Stromal Alterations in Multistep Hepatocarcinogenesis. PLoS One.

[17] Kim H, Choi GH, Na DC, Ahn EY, Kim GI, Lee JE (2011). Human hepatocellular carcinomas with "Stemness"-related marker expression: keratin 19 expression and a poor prognosis. Hepatology.

[18] Lee JI, Lee JW, Kim JM, Kim JK, Chung HJ, Kim YS (2012). Prognosis of hepatocellular carcinoma expressing cytokeratin 19: comparison with other liver cancers. World journal of gastroenterology.

[19] Chin L, Andersen JN, Futreal PA (2011). Cancer genomics: from discovery science to personalized medicine. Nature medicine.

[20] Greaves M, Maley CC (2012). Clonal evolution in cancer. Nature.

[21] Sidow A, Spies N (2015). Concepts in solid tumor evolution. Trends Genet.

[22] Alizadeh AA, Aranda V, Bardelli A, Blanpain C, Bock C, Borowski C (2015). Toward understanding and exploiting tumor heterogeneity. Nature medicine.

[23] Hanahan D, Weinberg RA (2011). Hallmarks of cancer: the next generation. Cell.

[24] Kan Z, Zheng H, Liu X, Li S, Barber TD, Gong Z (2013). Whole-genome sequencing identifies recurrent mutations in hepatocellular carcinoma. Genome Res.

[25] Vogelstein B, Papadopoulos N, Velculescu VE, Zhou S, Diaz LA Jr, Kinzler KW (2013). Cancer genome landscapes. Science.

[26] Schulze K, Imbeaud S, Letouze E, Alexandrov LB, Calderaro J, Rebouissou S (2015). Exome sequencing of hepatocellular carcinomas identifies new mutational signatures and potential therapeutic targets. Nat Genet.

[27] Li M, Zhao H, Zhang X, Wood LD, Anders RA, Choti MA (2011). Inactivating mutations of the chromatin remodeling gene ARID2 in hepatocellular carcinoma. Nat Genet.

[28] Fujimoto A, Totoki Y, Abe T, Boroevich KA, Hosoda F, Nguyen HH (2012). Whole-genome sequencing of liver cancers identifies etiological influences on mutation patterns and recurrent mutations in chromatin regulators. Nat Genet.

[29] Shibata T, Aburatani H (2014). Exploration of liver cancer genomes. Nat Rev Gastroenterol Hepatol.

[30] Shibata T, Arai Y, Totoki Y (2018). Molecular genomic landscapes of hepatobiliary cancer. Cancer science.

[31] Asati V, Mahapatra DK, Bharti SK (2016). PI3K/Akt/mTOR and Ras/Raf/MEK/ERK signaling pathways inhibitors as anticancer agents: Structural and pharmacological perspectives. European journal of medicinal chemistry.

[32] Zucman-Rossi J, Villanueva A, Nault JC, Llovet JM (2015). Genetic Landscape and Biomarkers of Hepatocellular Carcinoma. Gastroenterology.

[33] Houtgast EJ, Sima VM, Bertels K, Al-Ars Z (2018). Hardware acceleration of BWA-MEM genomic short read mapping for longer read lengths. Computational biology and chemistry.

[34] Brouard JS, Schenkel F, Marete A, Bissonnette N (2019). The GATK joint genotyping workflow is appropriate for calling variants in RNA-seq experiments. Journal of animal science and biotechnology.

[35] Cibulskis K, Lawrence MS, Carter SL, Sivachenko A, Jaffe D, Sougnez C (2013). Sensitive detection of somatic point mutations in impure and heterogeneous cancer samples. Nat Biotechnol.

[36] Wang K, Li M, Hakonarson H (2010). ANNOVAR: functional annotation of genetic variants from high-throughput sequencing data. Nucleic Acids Res.

[37] Blokzijl F, Janssen R, van Boxtel R, Cuppen E (2018). MutationalPatterns: comprehensive genome-wide analysis of mutational processes. Genome Med.

[38] Rosenthal R, McGranahan N, Herrero J, Taylor BS, Swanton C (2016). DeconstructSigs: delineating mutational processes in single tumors distinguishes DNA repair deficiencies and patterns of carcinoma evolution. Genome Biol.

[39] Gehring JS, Fischer B, Lawrence M, Huber W (2015). SomaticSignatures: inferring mutational signatures from single-nucleotide variants. Bioinformatics.

[40] Forbes SA, Beare D, Boutselakis H, Bamford S, Bindal N, Tate J (2017). COSMIC: somatic cancer genetics at high-resolution. Nucleic Acids Res.

[41] Talevich E, Shain AH, Botton T, Bastian BC (2016). CNVkit: Genome-Wide Copy Number Detection and Visualization from Targeted DNA Sequencing. PLoS Comput Biol.

[42] Bambury RM, Bhatt AS, Riester M, Pedamallu CS, Duke F, Bellmunt J (2015). DNA copy number analysis of metastatic urothelial carcinoma with comparison to primary tumors. BMC cancer.

[43] Kim D, Paggi JM, Park C, Bennett C, Salzberg SL (2019). Graph-based genome alignment and genotyping with HISAT2 and HISAT-genotype. Nat Biotechnol.

[44] Pertea M, Pertea GM, Antonescu CM, Chang TC, Mendell JT, Salzberg SL (2015). StringTie enables improved reconstruction of a transcriptome from RNA-seq reads. Nat Biotechnol.

[45] Love MI, Huber W, Anders S (2014). Moderated estimation of fold change and dispersion for RNA-seq data with DESeq2. Genome Biol.

[46] Boos DD, Stefanski LA (2011). P-Value Precision and Reproducibility. Am Stat.

[47] O'Brien SF, Osmond L, Yi QL (2015). How do I interpret a p value?. Transfusion.

[48] Wang R, Li J, Zhao Y, Li Y, Yin L (2018). Investigating the therapeutic potential and mechanism of curcumin in breast cancer based on RNA sequencing and bioinformatics analysis. Breast Cancer.

[49] Liu J, Li J, Li H, Li A, Liu B, Han L (2015). A comprehensive analysis of candidate genes and pathways in pancreatic cancer. Tumour Biol.

[50] Alajez NM (2016). Large-Scale Analysis of Gene Expression Data Reveals a Novel Gene Expression Signature Associated with Colorectal Cancer Distant Recurrence. PLoS One.

[51] Pazos AJ, Ventoso P, Martinez-Escauriaza R, Perez-Paralle ML, Blanco J, Trivino JC (2017). Transcriptional response after exposure to domoic acid-producing Pseudo-nitzschia in the digestive gland of the mussel Mytilus galloprovincialis. Toxicon.

[52] Padden J, Megger DA, Bracht T, Reis H, Ahrens M, Kohl M (2014). Identification of novel biomarker candidates for the immunohistochemical diagnosis of cholangiocellular carcinoma. Mol Cell Proteomics.

[53] Zhou Y, Zang Y, Yang Y, Xiang J, Chen Z (2019). Candidate genes involved in metastasis of colon cancer identified by integrated analysis. Cancer Med.

[54] Dennis G Jr, Sherman BT, Hosack DA, Yang J, Gao W, Lane HC (2003). DAVID: Database for Annotation, Visualization, and Integrated Discovery. Genome Biol.

[55] Wu J, Mao X, Cai T, Luo J, Wei L (2006). KOBAS server: a web-based platform for automated annotation and pathway identification. Nucleic Acids Res.

[56] Ashburner M, Ball CA, Blake JA, Botstein D, Butler H, Cherry JM (2000). Gene ontology: tool for the unification of biology. The Gene Ontology Consortium. Nat Genet.

[57] Kanehisa M (2002). The KEGG database. Novartis Foundation symposium.

[58] Franceschini A, Szklarczyk D, Frankild S, Kuhn M, Simonovic M, Roth A (2013). STRING v9.1: protein-protein interaction networks, with increased coverage and integration. Nucleic Acids Res.

[59] Smoot ME, Ono K, Ruscheinski J, Wang PL, Ideker T (2011). Cytoscape 2.8: new features for data integration and network visualization. Bioinformatics.

[60] Wang R, Wei B, Wei J, Tian Y, Du C (2017). Cysteine-rich 61-associated gene expression profile alterations in human glioma cells. Molecular medicine reports.

[61] Zhu Q, Sun Y, Zhou Q, He Q, Qian H (2018). Identification of key genes and pathways by bioinformatics analysis with TCGA RNA sequencing data in hepatocellular carcinoma. Mol Clin Oncol.

[62] Chin CH, Chen SH, Wu HH, Ho CW, Ko MT, Lin CY (2014). cytoHubba: identifying hub objects and sub-networks from complex interactome. BMC systems biology.

[63] Anaya J (2016). OncoLnc: linking TCGA survival data to mRNAs, miRNAs, and lncRNAs. PeerJ Computer Science.

[64] Dafni U (2011). Landmark analysis at the 25-year landmark point. Circulation Cardiovascular quality and outcomes.

[65] Li T, Fan J, Wang B, Traugh N, Chen Q, Liu JS (2017). TIMER: A Web Server for Comprehensive Analysis of Tumor-Infiltrating Immune Cells. Cancer research.

[66] Li B, Severson E, Pignon JC, Zhao H, Li T, Novak J (2016). Comprehensive analyses of tumor immunity: implications for cancer immunotherapy. Genome Biol.

[67] Li B, Li JZ (2014). A general framework for analyzing tumor subclonality using SNP array and DNA sequencing data. Genome Biol.

[68] de Klein A, van Kessel AG, Grosveld G, Bartram CR, Hagemeijer A, Bootsma D (1982). A cellular oncogene is translocated to the Philadelphia chromosome in chronic myelocytic leukaemia. Nature.

[69] Khatri A, Wang J, Pendergast AM (2016). Multifunctional Abl kinases in health and disease. Journal of cell science.

[70] Sandy P, Gostissa M, Fogal V, Cecco LD, Szalay K, Rooney RJ (2000). p53 is involved in the p120E4F-mediated growth arrest. Oncogene.

[71] Le Cam L, Linares LK, Paul C, Julien E, Lacroix M, Hatchi E (2006). E4F1 is an atypical ubiquitin ligase that modulates p53 effector functions independently of degradation. Cell.

[72] Ding C, Tang W, Fan X, Wang X, Wu H, Xu H (2018). Overexpression of PEAK1 contributes to epithelial-mesenchymal transition and tumor metastasis in lung cancer through modulating ERK1/2 and JAK2 signaling. Cell death & disease.

[73] Wang Y, Kelber JA, Tran Cao HS, Cantin GT, Lin R, Wang W (2010). Pseudopodium-enriched atypical kinase 1 regulates the cytoskeleton and cancer progression [corrected]. Proceedings of the National Academy of Sciences of the United States of America.

[74] Bristow JM, Reno TA, Jo M, Gonias SL, Klemke RL (2013). Dynamic phosphorylation of tyrosine 665 in pseudopodium-enriched atypical kinase 1 (PEAK1) is essential for the regulation of cell migration and focal adhesion turnover. The Journal of biological chemistry.

[75] Wang T, Kobayashi T, Takimoto R, Denes AE, Snyder EL, el-Deiry WS (2001). hADA3 is required for p53 activity. The EMBO journal.

[76] Nag A, Germaniuk-Kurowska A, Dimri M, Sassack MA, Gurumurthy CB, Gao Q (2007). An essential role of human Ada3 in p53 acetylation. The Journal of biological chemistry.

[77] Xiong Y, Piao W, Brinkman CC, Li L, Kulinski JM, Olivera A (2019). CD4 T cell sphingosine 1-phosphate receptor (S1PR)1 and S1PR4 and endothelial S1PR2 regulate afferent lymphatic migration. Sci Immunol.

[78] Xu Z, Liu L, Pan X, Wei K, Wei M, Liu L (2015). Serum Golgi protein 73 (GP73) is a diagnostic and prognostic marker of chronic HBV liver disease. Medicine.

[79] Jiao C, Cui L, Piao J, Qi Y, Yu Z (2018). Clinical significance and expression of serum Golgi protein 73 in primary hepatocellular carcinoma. Journal of cancer research and therapeutics.

[80] Chen MH, Jan YH, Chang PM, Chuang YJ, Yeh YC, Lei HJ (2013). Expression of GOLM1 correlates with prognosis in human hepatocellular carcinoma. Annals of surgical oncology.

[81] Connor F, Rayner TF, Aitken SJ, Feig C, Lukk M, Santoyo-Lopez J (2018). Mutational landscape of a chemically-induced mouse model of liver cancer. J Hepatol.

[82] Alexandrov LB, Jones PH, Wedge DC, Sale JE, Campbell PJ, Nik-Zainal S (2015). Clock-like mutational processes in human somatic cells. Nat Genet.

[83] Blokzijl F, de Ligt J, Jager M, Sasselli V, Roerink S, Sasaki N (2016). Tissue-specific mutation accumulation in human adult stem cells during life. Nature.

[84] Alexandrov LB, Kim J, Haradhvala NJ, Huang MN, Tian Ng AW, Wu Y (2020). The repertoire of mutational signatures in human cancer. Nature.

[85] Zhang Y, Kwok-Shing Ng P, Kucherlapati M, Chen F, Liu Y, Tsang YH (2017). A Pan-Cancer Proteogenomic Atlas of PI3K/AKT/mTOR Pathway Alterations. Cancer Cell.

[86] Pawlowski PH, Kaczanowski S, Zielenkiewicz P (2013). A kinetic model of the evolution of a protein interaction network. BMC Genomics.

[87] Das S, Meher PK, Rai A, Bhar LM, Mandal BN (2017). Statistical Approaches for Gene Selection, Hub Gene Identification and Module Interaction in Gene Co-Expression Network Analysis: An Application to Aluminum Stress in Soybean (Glycine max L.). PLoS One.

[88] Coma G, Pena R, Blanco J, Rosell A, Borras FE, Este JA (2006). Treatment of monocytes with interleukin (IL)-12 plus IL-18 stimulates survival, differentiation and the production of CXC chemokine ligands (CXCL)8, CXCL9 and CXCL10. Clin Exp Immunol.

[89] Nagtegaal ID, Odze RD, Klimstra D, Paradis V, Rugge M, Schirmacher P (2020). The 2019 WHO classification of tumours of the digestive system. Histopathology.

[90] Cong WM, Bu H, Chen J, Dong H, Zhu YY, Feng LH (2016). Practice guidelines for the pathological diagnosis of primary liver cancer: 2015 update. World journal of gastroenterology.

[91] Tombacz D, Maroti Z, Kalmar T, Csabai Z, Balazs Z, Takahashi S (2017). High-Coverage Whole-Exome Sequencing Identifies Candidate Genes for Suicide in Victims with Major Depressive Disorder. Sci Rep.

[92] Raychaudhuri P, Rooney R, Nevins JR (1987). Identification of an E1A-inducible cellular factor that interacts with regulatory sequences within the adenovirus E4 promoter. The EMBO journal.

[93] Huang L, Wen C, Yang X, Lou Q, Wang X, Che J (2018). PEAK1, acting as a tumor promoter in colorectal cancer, is regulated by the EGFR/KRas signaling axis and miR-181d. Cell death & disease.

[94] Kerr WG (2011). Inhibitor and activator: dual functions for SHIP in immunity and cancer. Annals of the New York Academy of Sciences.

[95] Yao M, Wang L, Leung PSC, Li Y, Liu S, Wang L (2018). The Clinical Significance of GP73 in Immunologically Mediated Chronic Liver Diseases: Experimental Data and Literature Review. Clin Rev Allergy Immunol.

[96] Alexiadis M, Rowley SM, Chu S, Leung DTH, Stewart CJR, Amarasinghe KC (2019). Mutational Landscape of Ovarian Adult Granulosa Cell Tumors from Whole Exome and Targeted TERT Promoter Sequencing. Mol Cancer Res.

[97] Awad D, Prattes M, Kofler L, Rossler I, Loibl M, Pertl M (2019). Inhibiting eukaryotic ribosome biogenesis. BMC Biol.

[98] Zhang Y, Baysac KC, Yee LF, Saporita AJ, Weber JD (2014). Elevated DDX21 regulates c-Jun activity and rRNA processing in human breast cancers. Breast Cancer Res.

[99] Nishida N, Kudo M (2017). Immunological Microenvironment of Hepatocellular Carcinoma and Its Clinical Implication. Oncology.

[100] Palucka AK, Coussens LM (2016). The Basis of Oncoimmunology. Cell.

[101] Lv L, Wang X, Ma T (2019). microRNA-944 inhibits the malignancy of hepatocellular carcinoma by directly targeting IGF-1R and deactivating the PI3K/Akt signaling pathway. Cancer Manag Res.

[102] Lin Z, Li S, Guo P, Wang L, Zheng L, Yan Z (2019). Columbamine suppresses hepatocellular carcinoma cells through down-regulation of PI3K/AKT, p38 and ERK1/2 MAPK signaling pathways. Life sciences.

[103] Wang L, Chen C, Feng S, Tian J (2018). TIPE-2 suppresses growth and aggressiveness of hepatocellular carcinoma cells through downregulation of the phosphoinositide 3-kinase/AKT signaling pathway. Molecular medicine reports.

[104] He XQ, Zhang YF, Yu JJ, Gan YY, Han NN, Zhang MX (2017). High expression of G-protein signaling modulator 2 in hepatocellular carcinoma facilitates tumor growth and metastasis by activating the PI3K/AKT signaling pathway. Tumour Biol.

[105] Lin H, Huang ZP, Liu J, Qiu Y, Tao YP, Wang MC (2018). MiR-494-3p promotes PI3K/AKT pathway hyperactivation and human hepatocellular carcinoma progression by targeting PTEN. Sci Rep.

[106] Huang JL, Cao SW, Ou QS, Yang B, Zheng SH, Tang J (2018). The long non-coding RNA PTTG3P promotes cell growth and metastasis via up-regulating PTTG1 and activating PI3K/AKT signaling in hepatocellular carcinoma. Mol Cancer.

[107] Li Q, Wang C, Wang Y, Sun L, Liu Z, Wang L (2018). HSCs-derived COMP drives hepatocellular carcinoma progression by activating MEK/ERK and PI3K/AKT signaling pathways. J Exp Clin Cancer Res.

[108] Pez F, Lopez A, Kim M, Wands JR, Caron de Fromentel C, Merle P (2013). Wnt signaling and hepatocarcinogenesis: molecular targets for the development of innovative anticancer drugs. J Hepatol.

[109] Dai Y, Liu L, Zeng T, Liang JZ, Song Y, Chen K (2018). Overexpression of MUC13, a Poor Prognostic Predictor, Promotes Cell Growth by Activating Wnt Signaling in Hepatocellular Carcinoma. Am J Pathol.

[110] Jiang C, Yu M, Xie X, Huang G, Peng Y, Ren D (2017). miR-217 targeting DKK1 promotes cancer stem cell properties via activation of the Wnt signaling pathway in hepatocellular carcinoma. Oncol Rep.

[111] Srivastava S, Thakkar B, Yeoh KG, Ho KY, Teh M, Soong R (2015). Expression of proteins associated with hypoxia and Wnt pathway activation is of prognostic significance in hepatocellular carcinoma. Virchows Archiv: an international journal of pathology.

[112] Wang Y, He L, Du Y, Zhu P, Huang G, Luo J (2015). The long noncoding RNA lncTCF7 promotes self-renewal of human liver cancer stem cells through activation of Wnt signaling. Cell stem cell.

[113] Nie X, Liu Y, Chen WD, Wang YD (2018). Interplay of miRNAs and Canonical Wnt Signaling Pathway in Hepatocellular Carcinoma. Front Pharmacol.

[114] Singh AK, Arya RK, Trivedi AK, Sanyal S, Baral R, Dormond O (2013). Chemokine receptor trio: CXCR3, CXCR4 and CXCR7 crosstalk via CXCL11 and CXCL12. Cytokine & growth factor reviews.

[115] Fukuda Y, Asaoka T, Eguchi H, Yokota Y, Kubo M, Kinoshita M (2020). Endogenous CXCL9 affects prognosis by regulating tumor-infiltrating natural killer cells in intrahepatic cholangiocarcinoma. Cancer science.

[116] Ding Q, Lu P, Xia Y, Ding S, Fan Y, Li X (2016). CXCL9: evidence and contradictions for its role in tumor progression. Cancer Med.

[117] Gorbachev AV, Kobayashi H, Kudo D, Tannenbaum CS, Finke JH, Shu S (2007). CXC chemokine ligand 9/monokine induced by IFN-gamma production by tumor cells is critical for T cell-mediated suppression of cutaneous tumors. Journal of immunology (Baltimore, Md: 1950).

[118] Wu Z, Huang X, Han X, Li Z, Zhu Q, Yan J (2016). The chemokine CXCL9 expression is associated with better prognosis for colorectal carcinoma patients. Biomedicine & pharmacotherapy = Biomedecine & pharmacotherapie.

[119] Pan J, Burdick MD, Belperio JA, Xue YY, Gerard C, Sharma S (2006). CXCR3/CXCR3 ligand biological axis impairs RENCA tumor growth by a mechanism of immunoangiostasis. Journal of immunology (Baltimore, Md: 1950).

[120] Fukuda Y, Asaoka T, Eguchi H, Yokota Y, Kubo M, Kinoshita M (2020). Endogenous CXCL9 affects prognosis by regulating tumor-infiltrating natural killer cells in intrahepatic cholangiocarcinoma. Cancer science.

[121] Ohira M, Ohdan H, Mitsuta H, Ishiyama K, Tanaka Y, Igarashi Y (2006). Adoptive transfer of TRAIL-expressing natural killer cells prevents recurrence of hepatocellular carcinoma after partial hepatectomy. Transplantation.

[122] Kunz M, Toksoy A, Goebeler M, Engelhardt E, Bröcker E, Gillitzer R (1999). Strong expression of the lymphoattractant C-X-C chemokine Mig is associated with heavy infiltration of T cells in human malignant melanoma. The Journal of pathology.

[123] Dangaj D, Bruand M, Grimm AJ, Ronet C, Barras D, Duttagupta PA (2019). Cooperation between Constitutive and Inducible Chemokines Enables T Cell Engraftment and Immune Attack in Solid Tumors. Cancer Cell.

[124] Bronger H, Singer J, Windmuller C, Reuning U, Zech D, Delbridge C (2016). CXCL9 and CXCL10 predict survival and are regulated by cyclooxygenase inhibition in advanced serous ovarian cancer. Br J Cancer.

[125] Mlecnik B, Tosolini M, Charoentong P, Kirilovsky A, Bindea G, Berger A (2010). Biomolecular network reconstruction identifies T-cell homing factors associated with survival in colorectal cancer. Gastroenterology.

[126] Denkert C, Loibl S, Noske A, Roller M, Müller BM, Komor M (2010). Tumor-associated lymphocytes as an independent predictor of response to neoadjuvant chemotherapy in breast cancer. Journal of clinical oncology: official journal of the American Society of Clinical Oncology.

