# Supplementary material for: Integrated Genomic and Transcriptomic Analysis reveals key genes for predicting dual-phenotype Hepatocellular Carcinoma Prognosis
Source: J Cancer. 2021 Mar 19;12(10):2993–3010. doi: 10.7150/jca.56005 (PMC8040886; doi:10.7150/jca.56005)
Supplement: Supplementary file 1 — Supplementary figures. [file jcav12p2993s1.pdf]

FigureS1

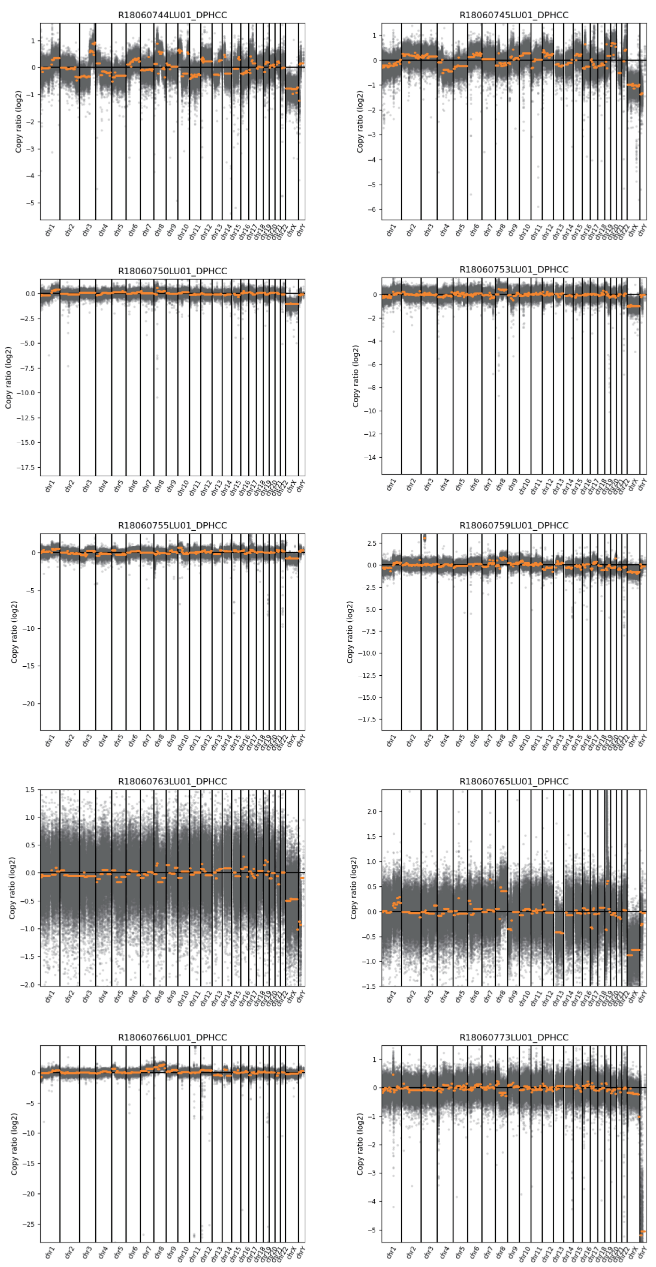

FigureS2

A

Cox regression results for CXCL9

Cancer	Cox Coefficient	P-Value	FDR Corrected	Rank	Median Expression	Mean Expression
BLCA	-0.068	3.50e-01	6.12e-01	9263	280.94	1428.1
BRCA	-0.149	8.90e-02	4.46e-01	3294	731.45	2443.76
CESC	-0.217	9.50e-02	4.17e-01	3690	826.68	2206.29
COAD	-0.115	2.60e-01	6.67e-01	6266	363.89	822.93
ESCA	0.053	7.00e-01	9.84e-01	11829	619.95	1820.94
GBM	-0.046	6.50e-01	9.52e-01	11382	59.39	122.35
HNSC	-0.139	4.90e-02	3.17e-01	2539	1179.58	3095.4
KIRC	-0.073	3.50e-01	4.81e-01	12072	1001.97	2005.38
KIRP	0.359	2.90e-02	1.02e-01	4640	69.05	257.37
LAML	-0.048	6.70e-01	8.82e-01	11501	4.06	33.22
LGG	0.273	5.50e-03	1.47e-02	6281	7.95	42.86
LIHC	-0.149	1.00e-01	3.16e-01	4924	317.95	954.96
LUAD	-0.065	4.10e-01	6.45e-01	10581	1092.15	2330.69
LUSC	-0.056	4.40e-01	8.46e-01	8720	970.11	1910.58
OV	-0.145	5.20e-02	6.48e-01	1338	369.21	942.74
PAAD	0.171	1.10e-01	2.81e-01	6540	270.78	848.39
READ	-0.05	8.30e-01	9.92e-01	13664	290.88	524.82
SARC	-0.345	1.60e-03	4.22e-02	605	195.1	1653.38
SKCM	-0.333	4.20e-07	1.02e-04	65	912.26	2948.55
STAD	-0.089	2.80e-01	6.59e-01	7064	1025.67	2752.54
UCEC	-0.201	4.70e-02	9.58e-01	783	159.09	755.74

B

Cox Proportional Hazard Model:

```
Model: Surv(LIHC) ~ Age + Gender + Stage + Neutrophil + Dendritic + B_cell + CD4_Tcell + CXCL9
339 patients with 112 dying
              coef     HR 95%CI_l   95%CI_u p.value sig
Age          0.010  1.010   0.995     1.025   0.193
gendermale  -0.098  0.906   0.608     1.350   0.628
stage2       0.261  1.299   0.784     2.151   0.310
stage3       0.910  2.485   1.597     3.869   0.000 ***
stage4       1.346  3.843   1.128    13.086   0.031   *
Neutrophil   1.340  3.817   0.000 33589.896   0.773
Dendritic    2.724 15.240   0.684   339.627   0.085   .
B_cell      -4.051  0.017   0.000    14.013   0.235
CD4_Tcell    2.121  8.337   0.066  1054.406   0.390
CXCL9       -0.155  0.856   0.739     0.992   0.039   *
Rsquare= 0.093 (max possible= 9.65e-01 )
Likelihood ratio test p= 2.45e-04
Wald test p= 9.38e-05
Score (logrank) test p= 3.04e-05
```

Figure S1. Copy number variants on Chromosomes of 10 DPHCC.

Figure S2. (A) Cox proportional risk regression analysis of the effect of CXCL9 expression on hepatocellular carcinoma patients using OncoLnc. (B) Cox proportional risk regression analysis of the effect of CXCL9 expression on hepatocellular carcinoma patients using TIMER.
